# Identification of Diabetic Patients through Clinical and Para-Clinical Features in Mexico: An Approach Using Deep Neural Networks

**DOI:** 10.3390/ijerph16030381

**Published:** 2019-01-29

**Authors:** Vanessa Alcalá-Rmz, Laura A. Zanella-Calzada, Carlos E. Galván-Tejada, Alejandra García-Hernández, Miguel Cruz, Adan Valladares-Salgado, Jorge I. Galván-Tejada, Hamurabi Gamboa-Rosales

**Affiliations:** 1 Unidad Académica de Ingeniería Eléctrica, Universidad Autónoma de Zacatecas, Jardín Juarez 147, Centro, Zacatecas 98000, Zac, Mexico; vdrar.06@uaz.edu.mx (V.A.-R.); lzanellac@uaz.edu.mx (L.A.Z.-C.); alegarcia@uaz.edu.mx (A.G.-H.); gatejo@uaz.edu.mx (J.I.G.-T.); hamurabigr@uaz.edu.mx(H.G.-R.); 2 Unidad de Investigación Médica en Bioquímica, Hospital de Especialidades, Centro Médico Nacional Siglo XXI, Instituto Mexicano del Seguro Social, Av. Cuauhtémoc 330, Col. Doctores, Del. Cuauhtémoc, Ciudad de México CP 06720, Mexico; miguel.cruzlo@imss.gob.mx (M.C.); adan.valladares@imss.gob.mx (A.V.-S.)

**Keywords:** type 2 diabetes, Artificial Neural Network, net reclassification improvement, computer-aided diagnosis, statistical analysis

## Abstract

Diabetes is a chronic and noncommunicable but preventable disease that is affecting the Mexican population at worrying levels, being the first place in prevalence worldwide. Early diabetes detection has become important to prevent other health conditions that involve low organ yield until the patient death. Based on this problem, this work proposes the architecture of an Artificial Neural Network (ANN) for the automated classification of healthy patients from diabetics patients. The analysis was performed used a set of 19 para-clinical features to determine the health status of the patients. The developed model was evaluated through a statistical analysis based on the calculation of the loss function, accuracy, area under the curve (AUC) and receiving operating characteristics (ROC) curve. The results obtained present statistically significant values, with accuracy of 0.94 and AUC values of 0.98. Based on these results, it is possible to conclude that the ANN implemented in this work can classify patients with presence of diabetes from controls with significant accuracy, presenting preliminary results for the development of a diagnostic tool that can be supportive for health specialists.

## 1. Introduction

According to the World Health Organization (WHO) and the International Diabetes Federation (IDF), the number of people with diabetes is increasing very quickly all over the world. Diabetes is one of the Noncommunicable Diseases (NCD), also known as chronic diseases, which are characterized by being of long duration and the result of a combination of different factors: genetic, environmental and behavior factors [1].

Diabetes is a major cause of death and disability worldwide and represents one of the greatest challenges of the 21st century for health and development. The NCD Risk Factor Collaboration (NCD-RisC) estimated that the number of people with diabetes quadrupled between 1980 and 2014. Age-standardized prevalence among adult men doubled during that time (from 4.3% to 9.0%), and age-standardized prevalence among adult women increased by 60% (from 5.0% to 7.9%) [2]. Moreover, diabetes has an important global connotation considering that, in 2017, 451 million people aged 18–99 years lived with diabetes and the number of people are predicted to rise to 693 million by 2045 [3,4].

Diabetes is defined as a group of metabolic diseases characterized by hyperglycemia resulting from defects in insulin secretion, insulin action, or both [5]. It consists of a complex disorder involving profound alterations in the metabolism of carbohydrates, fats and proteins [6].

In the scientific literature, this condition has been described under different terms, as a multifactorial and polygenic metabolic disorder, and its pathogenesis is influenced by diverse environmental and genetic risk factors [7]. Specifically, type 2 diabetes (T2D) is an expanding global health problem, closely linked to the epidemic of obesity [8].

Among the main effects of diabetes are long-term damage, dysfunction and failure of various organs, and vascular complications that shorten the life expectancy of those who suffer from this disease. For example, about 25% of persons with recent diagnosis have cardiovascular manifestations at the detection moment [9].

Therefore, there is an urgent need to implement population-based interventions that prevent diabetes and enhance its early detection [10].

There are several approaches to identify diabetes. For decades, the diagnosis of diabetes has been based on glucose criteria, either the FPG (Fasting Plasma Glucose) or the 2-h plasma glucose (2-h PG) value after a 75-g OGTT (Oral Glucose Tolerance Test) or A1C criteria [5,11].

Currently, algorithms based on computer-aided diagnosis (CADx) have been implemented for the diagnosis of diabetes through prediction models to know the future behavior of some data related to this disease.

Within these algorithms are found Artificial Neural Networks (ANN), which are based on mathematical models following the learning principle of artificial intelligence, as well as the natural response of the human neurons. ANNs are a nonlinear technique that integrates a set of variables through many data; for that reason, this procedure is useful for complex pattern recognition problems [12].

The implementation of ANN in this area is a tool that could be used by health services [13,14] because it gives information collected from diabetes cases and control cases (non-diabetic patients). Once the dataset has been analyzed, it is possible to improve the diabetes diagnosis, impacting in a positive way the health quality of the persons.

Carnimeo et al. [15] proposed the automatic detection of diabetic symptoms in retinal images by using a multilevel perceptron neural network. The network is trained using algorithms for evaluating the optimal global threshold that can minimize pixel classification errors. System performance is evaluated by an adequate index to provide a percentage measure in the detection of eye suspect regions based on neuro-fuzzy subsystem.

In addition, Cappon et al. [16] proposed the development of a tool based on ANN, optimizing and personalizing the calculation of the bolus calculation through continuous monitoring of glucose levels, obtaining useful and accessible information about patients that allows knowing if they have diabetes.

On the other hand, Chen et al. [17] proposed the 5G-Smart Diabetes system based on CADx, using different techniques of machine learning and big data to perform the analysis of patients suffering diabetes. In addition, the data sharing mechanism and personalized data analysis model for 5G-Smart Diabetes are presented in this work.

A continuing problem regarding diabetes despite its extensive study by different researchers is the difficulty of its early diagnosis and the identification of risks factors that may help to reduce the high incidence of this disease.

According to the above, in the present study, the objective was to analyze the relationship between anthropometric and biochemical features involved in the condition of diabetes. The main contribution of this work was to determine if a subject is presenting diabetes based in a set of specific features, which were analyzed through an ANN, obtaining a classification system that allows the identification of diabetic patients from controls.

Therefore, the novel aspect of this work was the analysis of this type of data through a tool of artificial intelligence looking for the relationship between the features used that allows automatically classifying subjects with presence of diabetes from controls to support the diagnosis of these patients through an automatic tool.

The rest of the paper is structured as follows. Section 2 describes the materials required for the development of this work as well as the methodology proposed, which consists in three main steps: data acquisition, data classification and validation. Section 3 presents the results obtained for each of the stages proposed. In Section 4, the results are discussed. In Section 5, the final conclusions are drawn.

## 2. Materials and Methods

The process performed for the classification between patients with presence of diabetes and patients with absence of this disease is presented in this section, as well as the description of the data and the validation of the results.

The methodology followed in this work consists in three main steps: (A) data acquisition; (B) data classification; and (C) validation.

Data were acquired from the general hospital “Centro Médico Siglo XXI” with information from Mexican patients. All subjects gave their informed consent for inclusion before they participated in the study. The study was conducted in accordance with the Declaration of Helsinki, and the protocol was approved by the Ethics Committee of “Instituto Mexicano del Seguro Social” and “Comisión Nacional de Investigación Científica” (R-2011-785-018). The patient data were then classified according to diabetes status. Finally, The ANN’s performance was evaluated, based on the objective of accurately classifying patients.

### 2.1. Subjects Description

The total number of patients contained in the dataset used for this work, is 1019, all Mexican, of which 499 correspond to non-diabetic patients (controls) and 520 to diabetic patients (cases). The age of the patients are between 35 and 65 years old, and 502 are female patients while 517 patients are male.

The criteria for the subjects to be part of the study were that the cases had less than five years of evolution and without other diseases. For the controls, the criterion was that they did not present any disease or metabolic syndrome. Pregnant women were excluded.

The diagnosis of the controls was made at the same time as the cases. The anthropometric and biochemical information was obtained at the same time the sample was taken and the sample was processed on the same day.

The glucose quantification was carried out for all participants in the overnight fasting of 12 h. The quantification was performed by the glucose oxidase method with the ILAB300 plus Instrument Laboratory, Bedford, MA, USA. The reference value was 70–100 mg/dL.

Finally, to ensure statistical significance, the sample was calculated with a 95% confidence level, with a 5% confidence interval over a estimated population size of 17,000,000 (estimated diabetic people in Mexico).

### 2.2. Features Description

In this work, 19 para-clinical features were analyzed, which are described in Table 1. They were used as input features to an ANN, while as output feature the health status based on the condition of diabetes of the patients was used.

### 2.3. Data Analysis

In this work, a data analysis based on a multivariate approach was performed, looking for the classification of patients according with their diabetes status. The input features represented the input layer of a deep ANN. Afterwards, a statistical validation was carried out to evaluate the results.

### 2.4. Data Preprocessing

The dataset was composed of 19 features, which were normalized through the Z-core method. This method transforms the data to a normal distribution with mean 0 and standard deviation 1. Z-score was used with the purpose of adequate data for the classification, because usually the data are not defined in the same numeric scale.

Once the dataset was normalized, it was randomly divided and balanced into two sets:

- The first one was the training set, which corresponded to the training stage, involving 70% of all data.
- The second was the test set, which corresponded to the test stage, involving the remaining 30%.

#### 2.4.1. Data Classification

The patients were classified based in an ANN approach according to their condition; the control patients were labeled with “0”, which are those who have not developed diabetes; and the case patients were labeled with “1”, which are those who have developed diabetes. This step was performed using a dense ANN that was specifically designed for this dataset, using the packages Tensorflow and Keras, for Python.

Tensorflow is an open-source software, symbolic math library focused in the dataflow programming based on the ranging of tasks, which is widely applied in different machine learning applications, such as ANN [18]. Keras is a high-level ANN API written in Python. It was created to perform fast experimentation using deep ANN, and it presents three maim characteristics: user-friendly, modular, and extensible [19].

ANNs can have different layers, which are composed by nodes or neurons. In general terms, an ANN tries to find a model that best describes the relationship between the input features and the output feature. The number of layers is modifiable as the number of neurons in each layer [20].

ANNs have three main elements:

- As mentioned above, an ANN has a set of connections that are called weights. The weights are the elements that connect the input signal with a neuron through the calculation of their product.
- The activation function affects the neurons, limiting the amplitude of the output with a finite value.
- An element summarizes the contributions of a weighted signal.

There are many types of layers but in this work two different layers were applied: a dense layer, which consists of a matrix of weights created by the layer and a vector of values called bias; and a dropout layer, which consists of randomly establishing a fractional rate, the main aim of this type of layer being to avoid an overfitting problem.

**Figure 1:**
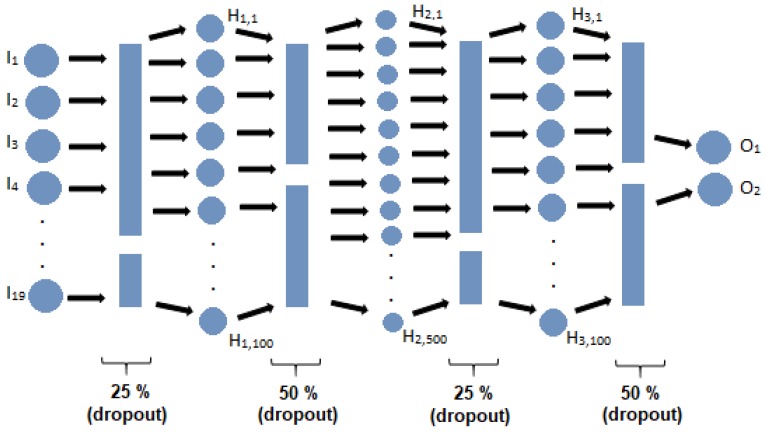
Graphic representation of the Artificial Neural Network (ANN) implemented.

The deep ANN designed for this work is shown in Figure 1, and the details of each layer are described below:

1. Input layer: 19 neurons (the data set has 19 features).
2. Dropout hidden layer: loss percentage of 25%.
3. Dense hidden layer: 100 neurons.
4. Dropout hidden layer: loss percentage of 50%.
5. Dense hidden layer: 500 neurons.
6. Dropout hidden layer: loss percentage of 25%.
7. Dense hidden layer: 100 neurons.
8. Dropout hidden layer: loss percentage of 50%.
9. Output layer: 2 neurons (control/diabetic).

For each dense layer, except the output dense layer, the activation function Rectified Linear Unit (ReLU) was added, which assigns 0 to the neurons that present a value lower than 0 and assigns the same value when this is equal or above 0. This function is calculated with Equation (1) [21].

(1) $$ReLu\left(z\right)=\left\{\begin{array}{cc} \mathrm{if}\;z<0 & 0\\ \mathrm{if}\;z\geq 0 & z \end{array}\right.$$

For the output layer, the activation function Softmax, also known as Normalized Exponential function, was added. Softmax is based on a general logistic function, compressing a vector of arbitrary values into a vector of values ranging [0, 1]. Equation (2) represents this function, where σ(*z*) refers to the *K*-dimensional vector, *z* [22].

(2) $$\sigma {\left(z\right)}_{j}=\frac{e^{z_{j}}}{\sum_{K=1}^{K}e^{z_{k}}},j=1,\ldots ,K$$

The optimization algorithm implemented was “Adam”, which is based in the stochastic gradient descent algorithm, using the average of the first and second moments of the gradients, adapting the learning parameter. In other words, this algorithm calculates the exponential moving average of the gradient and the square gradient, controlling the decay of that moving average [23].

The epoch number is a configurable value based on different parameters. For this work, there were established 100 epochs according to the results obtained through many tests with different number of epochs, where the best accuracy was reached using 100 epochs.

#### 2.4.2. Evaluation

For the evaluation stage, the loss function and accuracy were calculated on each epoch, and the receiving operating characteristics (ROC) curve was obtained with the average of the general behavior of the deep ANN.

When the value of the loss function goes down, it indicates that the model is fitting better to the data it is trying to model, looking for the global minimum, which represents the minimum error. In addition, this function optimizes the network feeding back with information of the system capacity [24]. The algorithm chosen to calculate the loss function in this work was “binary cross-entropy”, which is included in the Keras package for Python, and is able to calculate the cross-entropy parameter specifically in binary classification problems. This method is based on the Kullback–Leibler distance, which is calculated with Equation (3). This distance is a measure between two density functions *g* and *h*. Cross-entropy is an iterative method that generates a set of random values that are updated looking to generate more approximate values [25].

(3) $$D\left(g,h\right)=\int g\left(x\right)ln\frac{g(x)}{h(x)}\mu \left(dx\right)=\int g\left(x\right)lng\left(x\right)\mu \left(dx\right)-\int g\left(x\right)lnh\left(x\right)\mu \left(dx\right)$$

The accuracy is the parameter that calculates the average performance of the ANN based on the difference between the classification calculated and the real classification, as shown in Equation (4), calculating the accuracy as 1-error, where $V_{pred}$ is the classification value calculated and $V_{true}$ is the true classification value. It does not optimize the network but it obtains this value for each of the models, giving the option to select the model that presents the better performance [26].

(4) $$error=V_{pred}-V_{true}$$

The accuracy function selected for this work was “binary-accuracy” function from the Keras package. This function obtains the average accuracy based in the total predictions and it is used in binary classification problems specifically.

The ROC curve is a parameter used to measure the classification precision of the model, through the sensitivity and specificity. Sensitivity refers to the proportion of subjects with a positive condition that were correctly classified and it is calculated with Equation (5), where PPV is the positive predictive value, TP are the true positives and FP are the false positives [27].

(5) $$PPV=\frac{TP}{TP+FP}$$

Specificity refers to the proportion of subjects with a negative condition that were correctly classified and it is calculated with Equation (6), where NPV is the negative predictive value, TN are the true negatives and FN are the false negatives [27].

(6) $$NPV=\frac{TN}{TN+FN}$$

For each class, the ROC curves were obtained, as well as the ROC curve of the macro-average and the micro-average precision. The micro-average precision refers to the sum of the total true positives, false positives and false negatives for different sets, and it is calculated with Equation (7), where $TP_{1}$ are the true positives of one set, $TP_{2}$ are the true positives of a second set, $FP_{1}$ are the false positives of the first set and $FP_{2}$ are the false positives of the second [19].

(7) $$Micro-average=\frac{TP_{1}+TP_{2}}{TP_{1}+TP_{2}+FP_{1}+FP_{2}}$$

Finally, the macro-average precision is a value that calculates the average accuracy in different arbitrary sets and it is obtained with Equation (8), where $A_{1}$ is the average of one set and $A_{2}$ is the average of a second set.

(8) $$Macro-average=\frac{A_{1}+A_{2}}{2}$$

The implementation of this work was done with a laptop Toshiba Satellite S55T-B5233”, Intel Core i7-7500 U 2.70 GHz, 16 GB, 500 GB SSD, Ubuntu 16.4, 64-bit; and with Python version 2.7 (Toshiba, Tokio, Japan) [28].

All analyses were performed in Python 2.7.12, using the packages, Keras 2.1.5, Scipy 1.0.1, Pandas 0.22.0, Sklearn 0.191 and Tensorflow 1.7.0.

## 3. Results

The dataset was initially divided in two subsets, one for training containing 70% of the data (349 controls/364 cases), and one for testing containing 30% of the data (150 controls/156 cases).

The dataset used for the training of the deep ANN was evaluated at each epoch with the accuracy and loss function parameters. The number of epochs was tested with different values, as shown in Table 2, looking for the number of epochs that present the best result.

In addition, a comparison of the results using different type of layers, number of layers and number of neurons was made, which is shown in Table 3. The structure selected is presented in bold, which is characterized by a number of epochs of 100, in a deep ANN with nine layers, five dense layers and four dropout layers. The dense layers contained 19, 100, 500, 100 and 2 neurons, and the dropout layers presented 0.50, 0.25 and 0.50 percentages.

**Figure 2:**
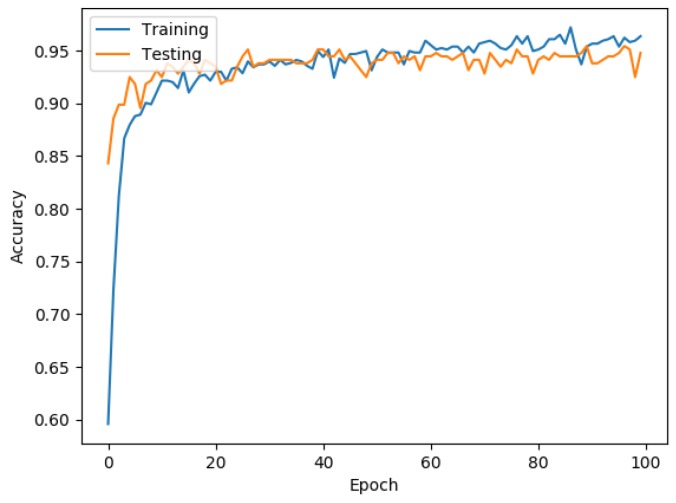
Accuracy behavior.

Figure 2 shows the behavior of the accuracy, where the blue line represents the training data, with an accuracy value of 0.96, and the orange line represents the testing data, with an accuracy value of 0.94.

**Figure 3:**
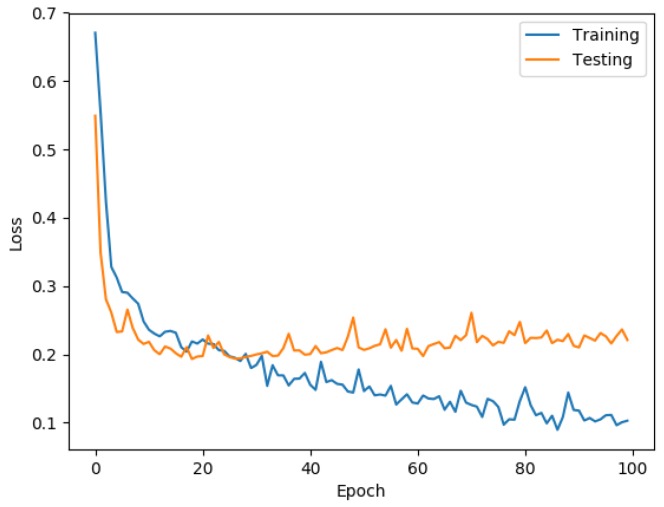
Loss function behavior.

Figure 3 shows the behavior of the loss function, where the blue line represents the training data, with a loss function value of 0.11, and the orange line represents the testing data, with a loss function value of 0.23.

Even when the accuracy and loss function values were obtained to measure the behavior of the classification of subjects, the accuracy is a parameter that may provide a better result than the real accuracy bias is present in the data. Due to this situation, the area under the curve (AUC) value was calculated to check that the results obtained were statistically significant.

**Figure 4:**
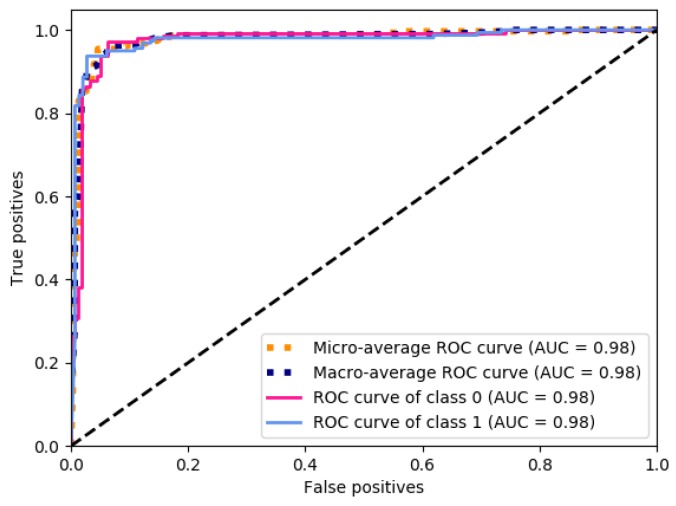
Receiving operating characteristics (ROC) curves obtained with the average performance of the ANN. AUC: area under the curve.

Figure 4 presents the ROC curves obtained based in the performance of the deep ANN. The ROC curve for the class 0 (control patients) is presented in pink, with an AUC value of 0.98. The ROC curve for the class 1 (case patients) is presented in light blue, with an AUC value of 0.98. The ROC curve calculated with the micro-average is presented in dotted orange, obtaining an AUC value of 0.98. Finally, the ROC curve calculated with the macro-average is presented in dotted dark blue, with an AUC value of 0.98.

## 4. Discussion

The dataset used in this work was classified into two subsets through an aleatory and balanced selection. One subset was used for the training of the deep ANN, which consisted of 100 epochs. This number of epochs was selected based in a comparison of different values, as shown in Table 2. Then, the ANN was optimized with the Adam algorithm to improve the behavior of the ANN through feedback.

The other subset was used for testing the ANN behavior, and it validated the results of the training stage through the accuracy and loss function values.

Figure 2 and Figure 3 show that the behaviors of the training data and testing data follow the same pattern, which indicates that the model obtained through the ANN became generalized with the learning process by being able to classify unknown data with the same performance as the known data.

It is important to remark that Figure 3 shows that the loss function is decreasing while the deep ANN is doing the training, as well as in the testing stage, which implies an approximation to the global minimum, while, in Figure 2, the behavior of the accuracy improves with the increase of the epochs for both datasets, indicating that both are improving their classification capacity in a similar proportion based on the learning.

The validation step allowed knowing that all ROC curves obtained, as presented in Figure 4, achieved statistically significant AUC values of around 0.98, which means that the ANN model presented a sensitivity—specificity rate that is able to classify the data with only 2% error.

Then, according to the results of the validation stage, the generalization of the model allowed significant values to be obtained for both classes, “0” and “1”, and for both measures, micro-average and macro-average.

It is important to mention that obtaining similar results in the micro-average and the macro-average represents great stability and robustness in this analysis, since the micro-average is calculated through the average of subsets of data and the macro-average is calculated using the complete dataset, implying that the data can be classified correctly regardless of how many are presented.

The positive results obtained are due to the features subjected to the ANN designed, as presented in Table 1, and the relationship between them. According to the literature, some of features are strongly related to diabetes disease and they can provide information to determine if a patient is prone to this condition. For example, the Body Mass Index (BMI), which is based on the weight and height, indicates the patient is prone to diabetes when it is ≥2.

In addition, compared to other works, this research presents higher values in the results of the obtained accuracy, as presented in Table 4.

On the other hand, one of the limitations of this work was related to the number of features used. It could be interesting to propose a dataset containing a greater number of features, looking for the improvement of the current results, to determine the main risk factors in the Mexican population.

In addition, it could be beneficial to create a device that implements the model developed to automate the diagnosis of diabetes and yield faster and more reliable results, supporting the initial diagnosis of the specialist.

It is important to mention that the implementation of the developed model does not need high computational cost, which is an advantage because it is not necessary to acquire any special hardware. Besides, for the development of this work, only free software was used, thus it does not imply any cost in licenses.

Finally, one of the points that most support the results is that the dataset contained the information of Mexican patients, which can help to improve the Mexican health through data-based tools developed with their own demography.

## 5. Conclusions

The results were validated through an evaluation step, being possible to conclude that the database was adequate for this work, demonstrating that is possible to classify persons with diabetes and persons who have not developed it, using the information of the 19 features previously mentioned.

The implementation of an ANN that was trained and tested with para-clinical data was to show the importance of having these types of data in the development of diabetes. Therefore, obtaining an accuracy of 0.96 allowed checking this hypothesis since the classification of cases from controls was correct 96% of the time.

The strong relationship between some features caused the good classification of persons according to the implemented model.The features used for this work represent the important risk factors to develop diabetes.

Because the dataset is based on Mexican people with presence and absence of diabetes, this work gives the advantage of knowing the important features to determinate this condition, and it is possible to create an auxiliary system that helps specialists to support their first diagnostic.

Based on these results, it is possible to conclude that these features present significant information for the classification of Mexican people with presence of diabetes from those with absence, which means that this information could be also used as a tool to support specialists for the preventive diagnosis and prediction of diabetes, helping to decrease the high incidence rate of this disease in Mexican population.

## Figures and Tables

**Table 1:** Features description.

Feature	Description
Age	Patient age at the time of analysis.
Gender	Patient gender (0—male/1—female).
Education	Studies concluded by the patient, (1—elementary school/2—secondary school, 3—high school/4—bachelor’s degree).
Weight	Patient weight in kilograms.
Height	Patient height in centimeters.
Waist	Patient waist perimeter in centimeters.
Hip Perimeter	Patient hip perimeter in centimeters.
BMI	Body Mass Index based on weight and height of a patient.
WHR	Waist Hip-Ratio based on the circumference of the waist to that of the hips.
SBP	Systolic Blood Pressure based on the pressure in the blood vessels when the heart beats.
DBP	Diastolic Blood Pressure based on the pressure in the blood vessels when the heart rests between beats.
Glucose	Blood glucose levels in terms of milligrams.
MMO Glucose	Blood glucose levels in terms of a molar concentration.
Insulin	Patient insulin in the blood.
HOMA	Homeostatic Model Assessment based on insulin resistance and beta-cell function.
Cholesterol	Fat-like substance that is found in all cells in the patient body.
LDL	Stands of low-density lipoprotein in the patient body.
HDL	Stands for high-density lipoprotein in the patient body.
TR	Triglycerides based on a type of fat (lipids) found in the patient body.
Output	Diabetes status (0—control/1—case).

**Table 2:** Accuracy and loss function values using different number of epochs.

Epochs	Accuracy	Loss Function	Processing Time (s)
10	0.93	0.21	1.59
50	0.94	0.20	5.12
100	0.96	0.21	9.35
150	0.94	0.23	13.96
200	0.93	0.25	18.23
300	0.93	0.30	27.18
500	0.94	0.29	44.12
1000	0.93	0.39	86.99

**Table 3:** Accuracy, loss function and processing time with different number of layers and neurons.

Layers Dense/Dropout	Neurons	Accuracy	Loss Function	Processing Time (s)
2/0	19 > 2	0.94	0.20	3.83
2/1	19 > 0.5 > 2	0.94	0.20	4.37
3/1	19 > 100 > 0.5 > 2	0.96	0.20	4.83
3/2	19 > 0.25 > 100 > 0.5 > 2	0.95	0.19	5.20
4/1	19 > 100 > 0.5 > 500 > 2	0.97	0.25	6.59
4/2	19 > 0.25 > 100 > 0.5 > 500 > 2	0.96	0.22	6.97
4/3	19 > 0.25 > 100 > 0.5 > 500 > 0.25 > 2	0.96	0.23	7.63
5/1	19 > 100 > 0.5 > 500 > 100 > 2	0.98	0.31	8.17
5/2	19 > 0.25 > 100 > 0.5 > 500 > 100 > 2	0.96	0.21	8.62
5/3	19 > 0.25 > 100 > 0.5 > 500 > 0.25 > 100 > 2	0.96	0.22	9.21
**5/4**	**19 > 0.25 > 100 > 0.5 > 500 > 0.25 > 100 > 0.5 > 2**	**0.96**	**0.21**	**9.50** ^1^

^1^ Structure selected for the Artificial Neural Network (ANN).

**Table 4:** Accuracy presented in related works.

Work	Description	Accuracy
Ndaba et al. [29]	Diabetes classification based on a regression ANN	86.00%
Soltani et al. [30]	Diabetes diagnosis based on a probabilistic ANN	89.56%
Sejdinović et al. [31]	Diabetes classification on an ANN	93.90%
Chen et al. [17]	Diabetes classification model based on boosting algorithms	95.30%
